# Parkinson’s disease: dopaminergic nerve cell model is consistent with experimental finding of increased extracellular transport of *α*-synuclein

**DOI:** 10.1186/1471-2202-14-136

**Published:** 2013-11-06

**Authors:** Finja Büchel, Sandra Saliger, Andreas Dräger, Stephanie Hoffmann, Clemens Wrzodek, Andreas Zell, Philipp J Kahle

**Affiliations:** 1 Center for Bioinformatics Tuebingen (ZBIT), University of Tuebingen, 72076 Tübingen, Germany; 2 Bioengineering Department, University of California, San Diego, La Jolla, CA 92093-0412, USA; 3 Laboratory of Functional Neurogenetics, Department of Neurodegeneration, Hertie Institute for Clinical Brain Research and German Center for Neurodegenerative Diseases, University of Tuebingen, 72076 Tübingen, Germany

**Keywords:** Parkinson’s disease, Dopaminergic nerve cell model, SBML model, Flux balance analysis

## Abstract

**Background:**

Parkinson’s disease is an age-related disease whose pathogenesis is not completely known. Animal models exist for investigating the disease but not all results can be easily transferred to humans. Therefore, mathematical or probabilistic models for the human disease are to be constructed *in silico* in order to predict specific processes within a cell, such as the dopamine metabolism and transport processes in a neuron.

**Results:**

We present a Systems Biology Markup Language (SBML) model of a whole dopaminergic nerve cell consisting of 139 reactions and 111 metabolites which includes, among others, the dopamine metabolism and transport, oxidative stress, aggregation of *α*-synuclein (*α*SYN), lysosomal and proteasomal degradation, and mitophagy. The predictive power of the model was investigated using flux balance analysis for the identification of steady model states. To this end, we performed six experiments: (i) investigation of the normal cell behavior, (ii) increase of O_2_, (iii) increase of ATP, (iv) influence of neurotoxins, (v) increase of *α*SYN in the cell, and (vi) increase of dopamine synthesis. The SBML model is available in the BioModels database with identifier MODEL1302200000.

**Conclusion:**

It is possible to simulate the normal behavior of an *in vivo* nerve cell with the developed model. We show that the model is sensitive for neurotoxins and oxidative stress. Further, an increased level of *α*SYN induces apoptosis and an increased flux of *α*SYN to the extracellular space was observed.

## Background

Parkinson’s disease (PD) is the second most frequent neurodegenerative disorder after Alzheimer’s disease. The mean age of onset is in the late 50s [1] and 1 % of the population older than 60 years is affected by PD [2]. The cell death of the dopaminergic neurons in the *substantia nigra* is responsible for typical disease symptoms: tremor, rigidity, and akinesia. The major genetic factor for PD is *α*-synuclein (*α*SYN). Protein aggregates that are the neuropathological hallmark of PD, called Lewy bodies (LBs), confirm *α*SYN as the principal component [3]. Besides *α*SYN, mutations in the genes encoding LRRK2 [4], parkin [5], PINK1 [6], and DJ-1 [7] are genetically linked to PD.

Until now, the underlying genetic and molecular mechanisms of PD have not been completely understood. Mittag *et al.* showed in their study that it is not possible to predict the disease risk for PD with top-validated single-nucleotide polymorphisms, although such a prediction is possible for type 1 diabetes [8]. Thus, in the case of PD, genetic markers alone cannot explain the disease outbreak. Therefore, more complex disease mechanisms must exist. While many animal models were developed for biological disease investigations, it is challenging to build one that elicits all aspects of the PD syndrome during aging. Some animal models exclusively reflect the symptoms of the disease or just a small fraction of them [9]. Further, not all findings of the animal models can be easily transferred to human beings, and it is not possible to investigate the molecular mechanisms of PD in a living human being. Therefore, mathematical models were developed to obtain insights into the cellular behavior [10,11]. A common method for the detailed investigation of these *in silico* models is flux balance analysis (FBA). It uses the reaction stoichiometry of the metabolic reactions to determine the most important cellular fluxes and cell steady states (where the cellular substance concentrations are in equilibrium) [12].

In this study, we apply a constraint-based modeling approach with the aim to derive a mathematical description of the basic dopaminergic nerve cell, whose predictions yield new insights of the disease mechanisms of PD. The quantitative computational dopaminergic nerve cell model has been stored in in the Systems Biology Markup Language (SBML). SBML is a widespread and machine-readable XML format that can be used for simulating, storing, and exchanging biological models [13]. It allows for a detailed description of metabolic reactions and is able to connect the model’s content to peer-reviewed databases. Our dopaminergic nerve cell model includes the complete dopamine (DA) synthesis, metabolism, and transport introduced by Best *et al.*[11]. Further, we model mitochondrial biogenesis and mitophagy [14,15], degradation processes of the lysosome and the proteasome [16], and reactions of the cell to reactive oxygen species (ROS) [17] and *α*SYN [18]. Additionally, the two genes HtrA2 and PINK1, both of which are involved in the mitochondrial stress response [19], are also integrated. The model is investigated using FBA by assigning different flux constraints and target functions. The experiments reproduce the normal behavior of an *in vivo* dopaminergic nerve cell at steady state and show that an increase of *α*SYN amplifies protein aggregation and leads to a drastically increased flux of *α*SYN to the extracellular space. This basic dopaminergic nerve cell model is publicly available at the BioModels database [20] with identifier MODEL1302200000 and can be further extended and investigated by other researchers to reveal insights of the underlying mechanism of PD.

## Results and discussion

### The dopaminergic nerve cell model

**Figure 1:**
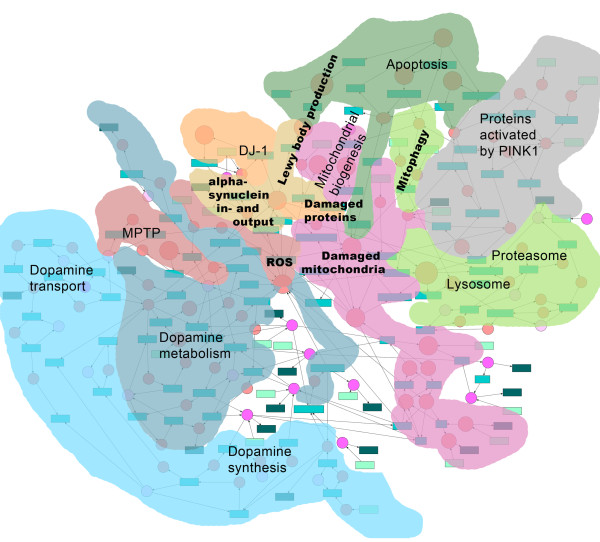
**Schema of the dopaminergic neve cell model.** The dopaminergic nerve cell model contains 111 metabolites (red and pink circles) and 139 reactions (rectangles). The different sub-models are labeled and indicated in different colors. Metabolites and reactions that are surrounded by multiple colors are assigned to multiple sub-models. The most strongly interconnected part of the model is in the middle and consists of *α*SYN, ROS, damaged proteins, and mitochondria.

The computational model of a dopaminergic nerve cell was developed and stored in SBML (see Additional file 1). It includes 111 metabolites and 139 reactions (see Additional files 2 and 3). The basis of the model comprises the publication by Best *et al.*[11], which mathematically describes the synthesis, metabolism, and the transport of DA in single dopaminergic neuron terminals (see sub-model 1-3). A detailed comparison of the model fluxes from Best *et al.* to our model fluxes is presented in Additional file 4. In addition, the model was enriched with information from previously published literature as well as with data from the databases PANTHER [21], TRANSPATH [22], STRING [23], Gene Ontology [24], and the Nature Pathway Interaction Database [25]. A schematic overview model is shown in Figure 1 and a detailed image is shown in Additional file 5.

The developed model consists of eleven sub-models, which are described here in more detail:

**Sub-model 1, 2, and 3 - DA synthesis, metabolism, and transport:** DA is synthesized from L-DOPA which is synthesized from L-tyrosine with the enzymes aromatic L-amino acid decarboxylase and tyrosine-hydroxylase (TH). The degradation of DA to homovanillic acid is catalyzed by monoamine oxidase (MAO) and the catechol-O-methyl transferase [26]. During this process, toxic side products can be built, such as salsolinol, which inhibits MAO and TH [27]. Besides these reactions, the model includes the release of DA vesicles as well as the re-uptake via the dopamine transporter (DAT). The interaction of DA with *α*SYN, which enhances the protein aggregation and reaction with ROS, is also included in the SBML model.

**Sub-model 4 - 1-methyl-4-phenyl-1,2,3,6-tetrahydropyridine(MPTP):** The neurotoxin MPTP is used to elicit PD symptoms in animal models (mouse or monkey). MPTP is the precursor of MPP^+^, which is imported into nigrostriatal DA neurons via DAT and irreversibly inhibits the mitochondrial respiration chain. Next, the uptake of MPTP causes neuronal cell death. We included MPTP in our model to represent several drugs that lead to drug-induced Parkinsonism [28].

**Sub-model 5 - Apoptosis:** The model includes two simple mechanisms that induce apoptosis, the first of which is the release of cytochrome C by defective mitochondria, which activates caspase-9. Second, we included the assumption that protein aggregates enhance the ROS production and induce apoptosis [3,29]. The process of apoptosis itself is not modeled in detail because the model focuses on the procedures that induce apoptosis.

**Sub-model 6 - Degradation:** The lysosome and the ubiquitin-proteasome-system (UPS) are responsible for the degradation of proteins or foreign substances in the cell. The basic degradation processes of nucleic acids, polysaccharides, proteins, and lipids by the lysosome are contained in the model. The UPS is modeled in detail to investigate the influence of the E3-ligase Parkin on the ubiquitylation of misfolded proteins [16].

**Sub-model 7 -*α*** **SYN and LB formation:***α*SYN, ubiquitin, neurofilaments, and other proteins build the PD characteristic protein aggregates [18]. The nerve cell model includes the *α*SYN aggregation by the interaction of *α*SYN with DA and the aggregation enhanced by the interaction with ROS. Further, the aggregation inhibition by DJ-1 (see sub-model 8) is modeled as well as the inhibition of the mitochondrial fusion by DJ-1 [30].

**Sub-model 8 - The chaperone DJ-1:** DJ-1 is the product of the PARK7 gene. Defects in this protein are causal for an autosomal-recessive form of PD [7] and DJ-1 knock-out leads to increased ROS production and the elongation of the mitochondria [31]. DJ-1 has a redox-sensitive chaperone function, is an indicator for oxidative stress, and is able to protect neurons against this form of stress [32]. The model includes these protection effects against oxidative stress as well as the function to inhibit the aggregation and toxic influence of *α*SYN [14,15].

**Sub-model 9 - Mitochondrial quality control:** Steady fusion and fission processes maintain the morphology and the membrane potential of mitochondria. If a mitochondrion is defective it can be repaired through three different quality control processes: (i) molecular quality control, (ii) organella quality control, and (iii) cellular quality control [33]. The first mechanism involves HtrA1/Omi, PINK1, and TRAP1 and leads to mitochondrial fission and conversely OPA1 mediated fusion. In the second mechanism, the mitochondrion is removed by autophagy involving PINK1 and Parkin, and VDAC1 or mitofusin [34]. The third mechanism starts if the other mechanisms fail. Then, the mitochondrion releases pro-apoptotic factors and the cell dies. The model includes the respiration chain with ATP production, the three repair mechanisms, and the production of ROS by defective mitochondria.

**Sub-model 10 - Protein phosphorylation by PINK1:** The model also represents the phosphorylation of Parkin, HtrA2/Omi, and TRAP1 by PINK1 [15]. Parkin inhibits, similar to phosphorylated TRAP1, the release of cytochrome C and consequently, Parkin and TRAP prevent apoptosis [35-37]. Additionally, Parkin stimulates the repair of defective mitochondria [38] and TRAP1 inhibits mitochondrial protein misfolding (see sub-model 9) [39].

Phosphorylation by PINK1 was suggested to activate the proteolytic activity of HtrA2/Omi and confer some resistance to mitochondrial stress [40].

**Sub-model 11 - molecular units of currency:** This part includes the molecular units of currency, namely O_2_, H_2_O, NAD^+^, NADH, ${\text{Fe}}_{3}^{+}$, ${\text{Fe}}_{2}^{+}$, ADP + Pi, and ATP. For the FBA, these substances are allowed to flow in and out of the model within the defined ranges.

We restricted our model to autonomous cell events. The LRRK2 gene, which is the most common autosomal-dominant gene and an important genetic cause for sporadic PD, was not included in the core model because its complete function remains unknown [41]. It is recently believed that LRKK2 is involved in immune processes [42].

The nerve cell model was further annotated using methods from the application KEGGtranslator [43,44] and published in the BioModels database. It is available with identifier MODEL1302200000 and as Additional file 1.

### Model analysis

We performed six *in silico* experiments: (i) investigation of the normal cell behavior, (ii) increase of O_2_, (iii) increase of ATP, (iv) influence of neurotoxins, (v) increase of *α*SYN in the cell, and (vi) increase of dopamine synthesis (see Table 1). For each experiment at least two flux balance analyses were performed with different target functions and varying input fluxes. The target functions minimal apoptosis (minApo) and maximal degradation (maxDeg) were always used for the analysis but maximal apoptosis (maxApo) and minimal degradation (minDeg) were selected only once for the initial model investigation. The experiments are presented in detail below.

**Table 1:** Parameters and target functions of the flux balance analyses

**Experiment**	**Performed FBA with the**	**Flux range**
	**corresponding target function**	**[*μ*** **M·h**^**−1**^**]**
Normal nerve cell	minApo, maxApo, minDeg, maxDeg	-
O_2_	minApo, maxDeg	0-100
ATP	minApo, maxDeg	0-1000
Drug influence (MPTP)	minApo, maxDeg	0-100
Increased *α*SYN flux	minApo, maxDeg	0-100
Increased tyrosine flux	minApo, maxDeg	0-100

Six experiments were performed. For each experiment, at least two different FBAs were performed with different target functions and variable flux ranges that are denoted in the last table column. Abbreviations: minimizing the output-reaction of the apoptosis (minApo), maximizing the output-reaction of the apoptosis (maxApo), minimizing the output-reaction of the degradation (minDeg), maximizing the output-reaction of the degradation (maxDeg), 1-methyl-4-phenyl-1,2,3,6-tetrahydropyridine (MPTP).

#### Conversion of the healthy cell to a diseased cell

In the first experiment, we investigated the model of a healthy dopaminergic nerve cell to determine the normal flux ranges. Compared to the model by Best *et al.* the resulting model from our study contains more reactions with cytosolic dopamine as a substrate. Thus, it was necessary to adapt the suggested initial flux bounds from Best *et al.* (see Additional file 4).

**Figure 2:**
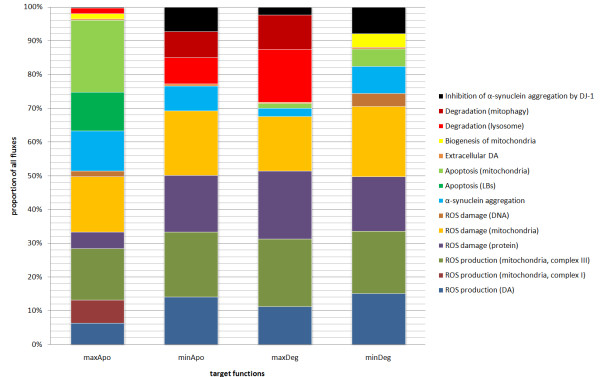
**Comparison of the basic-model fluxes.** The diagram shows the results of four flux balance analyses of the basic dopaminergic nerve cell. As target functions, the maximization of apoptosis (maxApo), the minimization of apoptosis (minApo), the maximization of degradation (maxDeg), and the minimization of degradation (minDeg) were optimized, respectively. Interestingly, the mitochondrial production of ROS by complex I and the apoptosis initiated by LB can be observed only in the maxApo analysis. Mitophagy is performed in minApo and maxDeg, whereas the biogenesis is only performed in maxApo and minDeg. Degradation processes appear mainly in the minApo and maxDeg analysis but are extremely diminished or nonexistent in the maxApo and minDeg analyses.

In aged cells, there are more damaged proteins and ROS, which need to be degraded [17]. A healthy nerve cell is able to degrade damaged proteins and initiates apoptosis only if there is no alternative [17,45]. This may also reflect the cellular behavior of neurons during the disease outbreak of PD. For that reason we selected apoptosis and degradation fluxes as target functions for the FBA, i.e., those functions are gradually maximized during our analyses. The results are depicted in Figure 2 and Table 2. The initiation and the apoptosis itself are very complex mechanisms [46] and constitute fundamental parts of the model. We assume that the optimization of the minApo and maxDeg target analyses best reflects the normal neuronal cell behavior because cell death is a late event in neurodegenerative diseases, and before initiating apoptosis the cell degrades damaged cell products [47]. We examined this assumption by minimizing apoptotic processes in the FBA and observed increased degradation and increased ROS elimination processes but no apoptosis. Apoptosis appears when we maximize the degradation processes and it even increases during the minDeg analysis. During this analysis, we also observed apoptosis induced by mitochondria. This flux is also increased in the maxDeg experiment. In the maxApo analysis, DJ-1 activity and degradation processes are nearly missing. This is due to the underlying optimization algorithm of the FBA, which forces apoptotic processes. Therefore, we used minApo and maxDeg as target functions for the following experiments.

**Table 2:** Fluxes of the basic model

	**Fluxes in *μ*** **M·h**^**−1**^			
	**Basic model**			
**Reaction (group)**	**maxApo**	**minApo**	**maxDeg**	**minDeg**
ROS production (DA)	19.00	19.00	19.56	19.00
ROS production (mitochondria, complex I)	21.15	0.00	0.00	0.00
ROS production (mitochondria, complex III)	46.04	26.05	35.06	23.35
ROS elimination (DJ-1)	0.00	0.00	0.00	0.00
ROS damage (protein)	14.98	22.73	35.17	20.31
ROS damage (mitochondria)	50.00	25.92	28.23	26.21
ROS damage (DNA)	5.00	0.00	0.00	5.00
Apoptosis (LBs)	35.00	0.00	0.00	0.00
Apoptosis (mitochondria)	65.00	0.00	2.40	6.47
Degradation (lysosome)	4.98	10.58	27.27	0.00
Degradation (proteasome)	0.00	0.00	0.00	0.00
Degradation (mitophagy)	0.00	10.43	17.73	0.00
Biogenesis of mitochondria	5.00	0.00	0.00	5.00
Extracellular DA	0.80	0.80	0.80	0.80
*α*SYN aggregation	36.00	9.89	4.24	10.00
*α*SYN output reaction	0.00	0.00	0.00	0.00
Toxic effect of *α*SYN aggregates	0.00	0.00	0.00	0.00
Inhibition of *α*SYN aggregation by DJ-1	1.00	9.89	4.24	10.00

The table shows the FBA results of the basic dopaminergic nerve cell experiment. Four different target functions were selected for the simulation: (i) minApo, (ii) maxApo, (iii) minDeg, (iv) maxDeg. The table includes three reactions groups: ‘ROS production (dopamine)’, ‘ *α*SYN aggregation’ and ‘Toxic effects of *α*SYN aggregates’. ‘ROS production (dopamine)’ comprise all reactions of the dopamine synthesis and metabolism, where ROS or ROS-like metabolites are produced. These reactions are *DOPALlikeROS*, *SalsolinollikeROS*, *DopaminesyntheseROSout*, *DOPALSynthesis2*, and *HVAldehydSynthesis2*. ‘ *α*SYN aggregation’ includes the reaction of DA and *α*SYN (*SNCADopamineAggregation*), the reaction of ROS and *α*SYN (*SNCAROSAggregation*), and the increased *α*SYN expression (*SNCAOverexpression*). Finally, ‘Toxic effects of *α*SYN aggregates’ contains the reaction *MitochondriaDamageSNCAAggregates* and *isdamagedProtein*. For each reaction group, the reaction fluxes are added up to obtain a better overview of the corresponding topic.

#### O_2_ experiment

**Figure 3:**
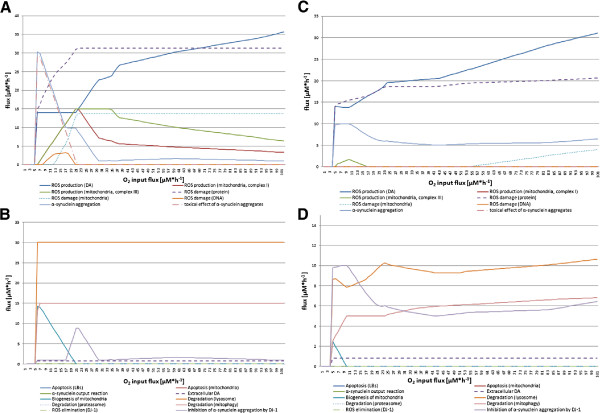
**Cellular behavior with increasing O**_**2**_**.** The four diagrams show the flux changes while the O_2_ input flux is increased from 0 to 100 *μ*M·h^−1^. Sub-diagram **A** and **B** depict the results of the FBA with target function maxDeg. Sub-diagram **C** and **D** display the results of the FBA with target function minApo. Sub-diagram **A** and **C** summarize all fluxes that produce ROS and/or cause damage in the cell. In contrast, sub-diagram **B** and **D** contain all fluxes that are considered to be responses or consequences of fluxes shown in sub-diagram **A** to retain the normal cellular function.

In addition to the previous experiment, the input reaction of O_2_ is included in the optimization function of the FBA. For both flux balance analyses with target functions minApo and maxDeg, no flux is observed between 0 *μ*M·h^−1^ and 4 *μ*M·h^−1^O_2_ (see Figure 3). At an input flux between 4 and 21 *μ*M·h^−1^ the ROS production during the DA synthesis rises, as does the production of ROS by complex III of the respiratory chain increase. This rise is higher in the maxDeg experiment than in the minApo experiment. Futher, an immediate increase of the *α*SYN aggregation process can be observed in the maxDeg experiment, but it falls off at an O_2_ input flux of 21 *μ*M·h^−1^. In summary, this experiment shows that at an O_2_ input flux of 30 *μ*M·h^−1^ or higher the cell is in a stable condition and no apoptosis is initiated.

#### ATP experiment

**Figure 4:**
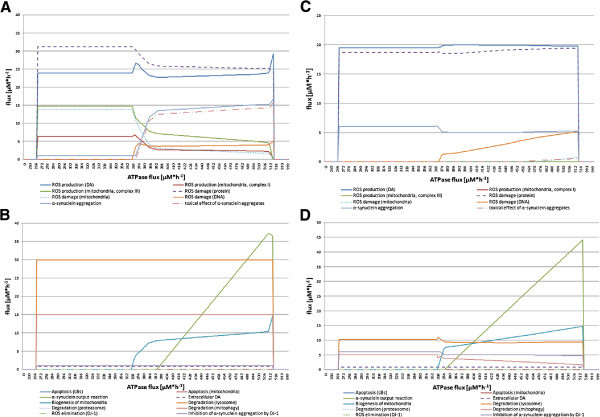
**Cellular behavior with increasing ATP production.** The four diagrams show the flux changes while the ATPase input flux is increased from 0 to 1000 *μ*M·h^−1^. Sub-diagram **A** and **B** depict the results of the FBA with target function maxDeg. Sub-diagram **C** and **D** display the results of the FBA with target function minApo. Sub-diagram **A** and **C** summarize all fluxes that produce ROS and/or cause damage in the cell. In contrast, sub-diagram **B** and **D** contain all fluxes that are considered responses or consequences of fluxes shown in sub-diagram A to retain the normal cellular function.

In this experiment the energy consumption of the system is investigated (see Figure 4). The resulting steady state fluxes show that an ATP flux of at least 266 *μ*M·h^−1^ up to 512 *μ*M·h^−1^ is required for the cellular processes. We observed that the ATP production correlates with the SNCA input, because this reaction is coupled to the ATP consumption in the model. Thus, at an ATP input flux of 370 *μ*M·h^−1^ the cell produces too much ATP. Initially, the ATP surplus is transported out of the cell. But with increasing ATP, more ATP consuming reactions, such as *α*SYN input and output reactions, are boosted in order to gain a solution to the FBA. In conclusion, the model’s optimal behavior is achieved between an ATP flux of 266 *μ*M·h^−1^ and 368 *μ*M·h^−1^.

#### MPTP experiment

**Figure 5:**
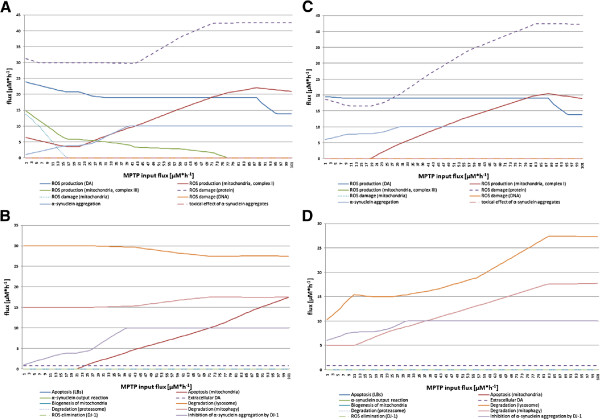
**Cellular behavior after cell penetration with MPTP.** The four diagrams show the flux changes while the MPTP input flux is increased from 0 to 100 *μ*M·h^−1^. Sub-diagram **A** and **B** depict the results of the FBA with target function maxDeg. Sub-diagram **C** and **D** display the results of the FBA with target function minApo. Sub-diagram **A** and **C** summarize all fluxes that produce ROS and/or cause damage in the cell. In contrast, sub-diagram **B** and **D** contain all fluxes that are considered responses or consequences of fluxes shown in sub-diagram **A** to retain the normal cellular function.

MPTP is an established neurotoxin to induce PD [48]. In this experiment, we investigated whether the model behaves like a normal dopaminergic nerve cell. The results of this experiment are shown in Figure 5. In both FBA experiments with target function minApo and maxDeg, the ROS production fluxes of complex I of the respiratory chain increases steadily. In the minApo experiment, the degradation flux of the lysosome increases in parallel. The same behavior can be observed for the aggregation of *α*SYN and the inhibition of the aggregation process by DJ-1. During the FBA with target function minApo, no apoptosis is observed which is due to the FBA simulation behavior. However, with the maxDeg FBA, apoptosis starts at an MPTP input flux of 23 *μ*M·h^−1^, which is initiated by the mitochondria.

#### *α*-synuclein experiment

**Figure 6:**
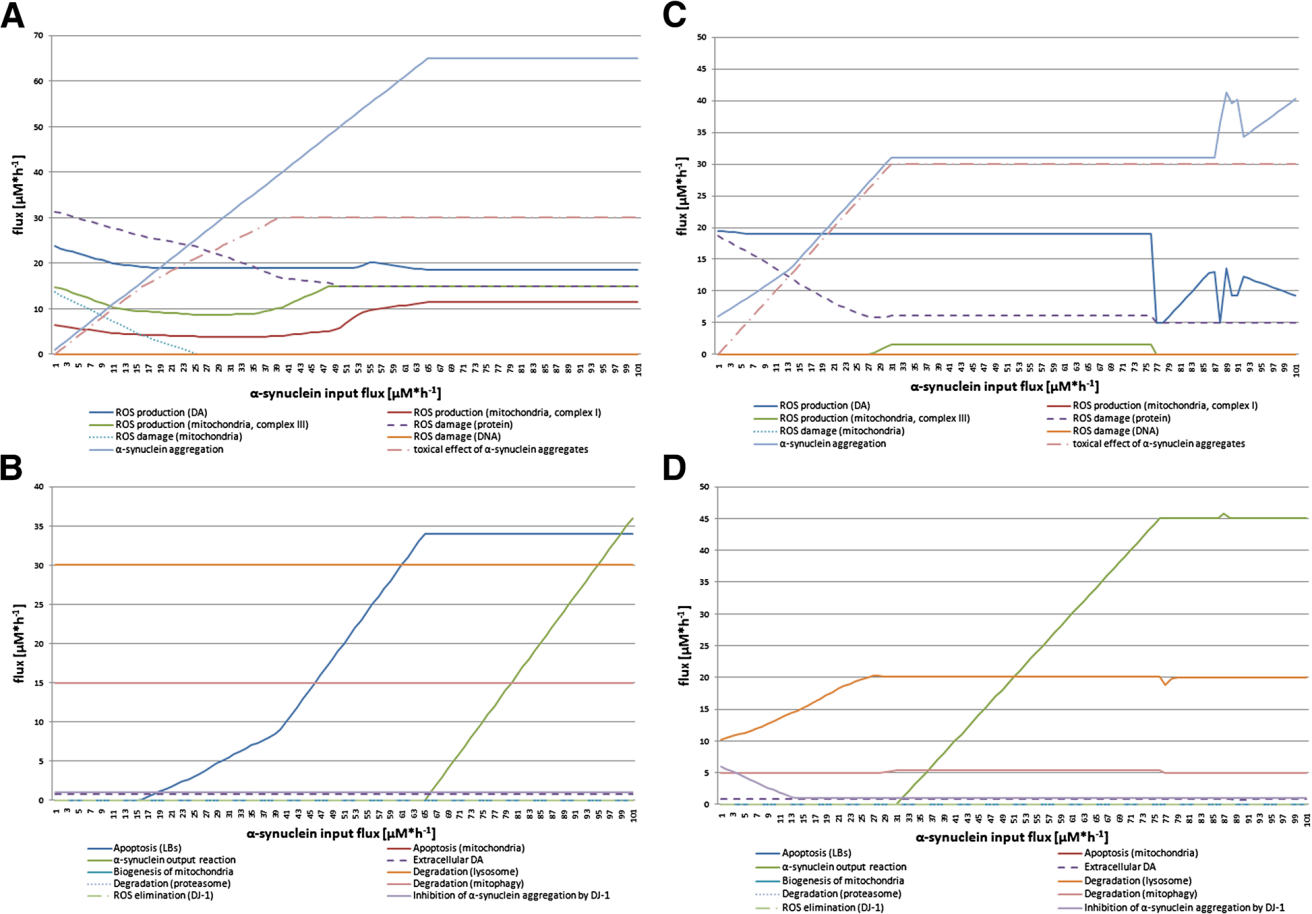
**Effect of increased*α*SYN flux in the dopaminergic nerve cell.** The four diagrams show the flux changes while the *α*SYN input flux is increased from 0 to 100 *μ*M·h^−1^. Sub-diagram **A** and **B** depict the results of the FBA with target function maxDeg. Sub-diagram **C** and **D** display the results of the FBA with target function minApo. Sub-diagram **A** and **C** summarize all fluxes that produce ROS and/or cause damage in the cell. In contrast, sub-diagram **B** and **D** contain all fluxes that are considered responses or consequences of fluxes shown in sub-diagram **A** to retain the normal cellular function.

Here, we investigated the influence of the *α*SYN concentration in the dopaminergic nerve cell (Figure 6) by performing two FBA simulations with minApo and maxDeg, respectively, as target functions. Point mutations in SNCA lead to a higher propensity to aggregate as Lewy bodies [49,50]. To simulate this behavior, the *α*SYN flux was steadily increased from 0 to 100 *μ*M·h^−1^ (see Figure 6). The increased aggregation tendency could be confirmed by our model, where the *α*SYN aggregation continuously rises from 1 *μ*M·h^−1^ to 31 *μ*M·h^−1^. In parallel, the toxic effects of *α*SYN aggregates increase (see Figure 6A). In contrast, the ROS production of DA metabolism decreases as well as the damage of ROS for proteins in both experiments. In the minApo experiment, a steady inhibition of *α*SYN aggregation by DJ-1 with a flux of 8 *μ*M·h^−1^ exists, but it decreases at an *α*SYN input flux of 13 *μ*M·h^−1^.

In conclusion, there is no essential contribution of DJ-1 towards inhibiting *α*SYN aggregation in the two FBA experiments. For an *α*SYN input flux higher than 78 *μ*M·h^−1^, the FBA, with respect to the target function minApo, shows unexpected fluctuations in the ROS production flux of the DA synthesis and in the *α*SYN aggregation flux (see Figure 6C). This observation can be explained by the defined flux ranges of the DA synthesis reactions. Compared to all other model constraints, these bounds are defined in a very narrow range due to the knowledge that some reactions proceed slower than others. Therefore, reactions with larger flux ranges need to be divided, and this separation causes the observed behavior. Another interesting effect is that the *α*SYN output flux that transports *α*SYN to the extracellular space increases simultaneously with the rise of the *α*SYN input flux with a small peak at a flux of 87 *μ*M·h^−1^. This behavior stabilizes at an *α*SYN input flux of 65 *μ*M·h^−1^ for maxDeg and at a flux of 75 *μ*M·h^−1^ for minApo FBA. Apoptosis is only observed in the maxDeg FBA and is initiated by the aggregation of *α*SYN.

#### DA experiment

**Figure 7:**
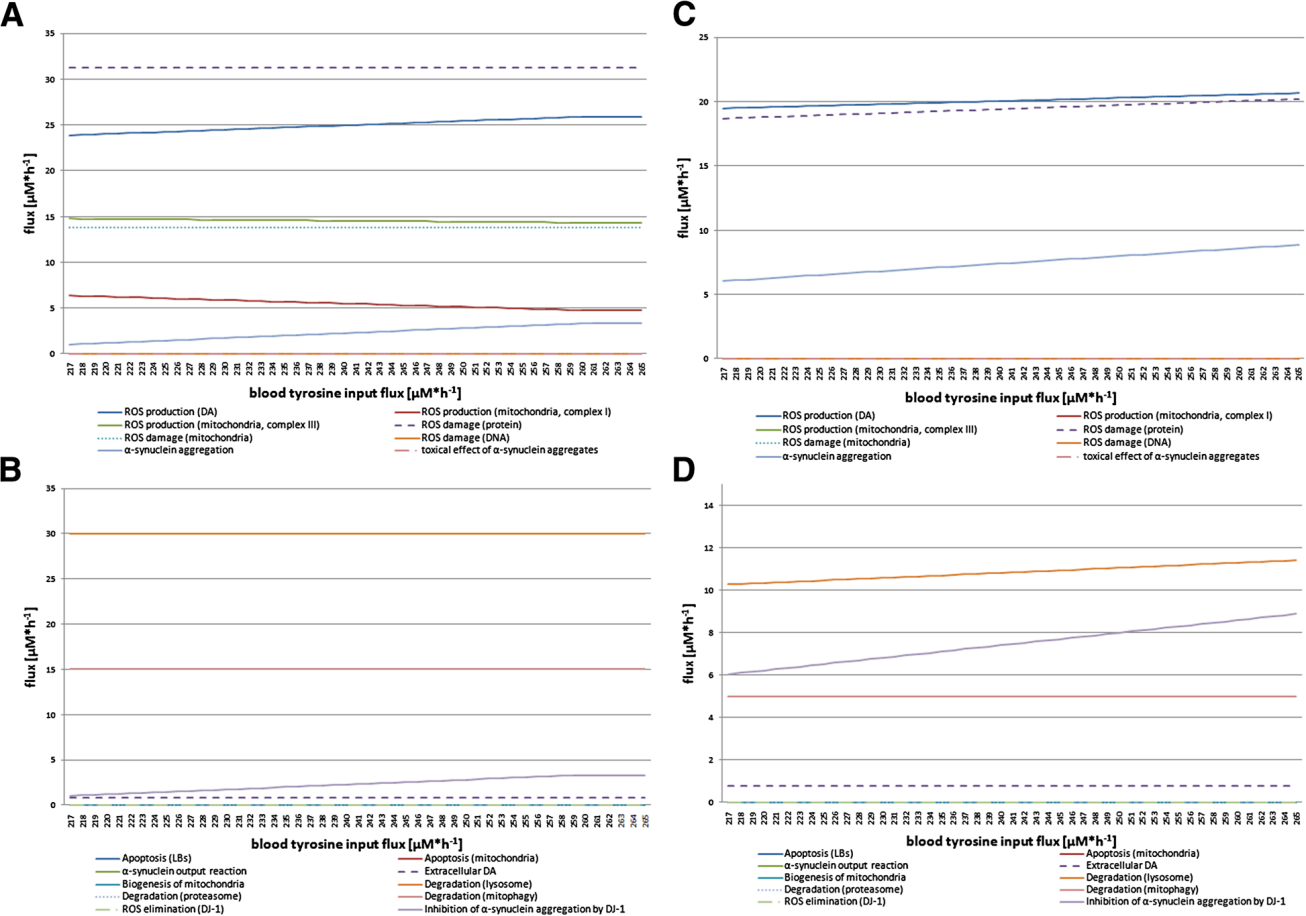
**Investigation of an increased DA amount in the nerve cell.** The four diagrams show the flux changes while the tyrosine input flux is increased from 0 to 100 *μ*M·h^−1^. Sub-diagram **A** and **B** depict the results of the FBA with target function maxDeg. Sub-diagram **C** and **D** display the results of the FBA with target function minApo. The tyrosine input reaction acts as precursor flux for the DA synthesis and metabolism in the nerve cell model. If this flux is increased, the DA content in the cell rises. Sub-diagram **A** and **C** summarize all fluxes that produce ROS and/or cause damage in the cell. In contrast, sub-diagram **B** and **D** contain all fluxes that are considered responses or consequences of fluxes shown in sub-diagram **A** to retain the normal cellular function.

Galvin suggests that 3,4-dihydroxyphenylacetaldehyde, a metabolite of the DA metabolism, may influence the development of oxidative stress and the interaction with *α*SYN [51]. In the last experiment, we increased the tyrosine input flux from 0 to 100 *μ*M·h^−1^ because this is the precursor flux to induce the DA metabolism (see Figure 7). In this experiment, we could not observe any apoptotic processes during the FBA with target function maxDeg or minApo. There is a slight occurrence of *α*SYN aggregation in both flux balance analyses, but an increased lysosomal degradation at the same time. We observed a constant ROS production due to the increased DA production and an increased ROS production by complex I in the respiratory chain, which affects damaged proteins in this experiment. We could not observe any damage of mitochondria or DNA.

In summary, we observed that a higher DA cell concentration is not causal for PD but it can positively stimulate its development by producing ROS.

## Conclusion

In this study, a basic dopaminergic nerve cell model was generated and made available in the BioModels database. This model contains DA synthesis, metabolism and release, the respiration chain and anti-oxidative defense mechanisms, the degradation system represented by mitophagy, the lysosome and the proteasome, as well as the apoptosis pathway. Besides these basic cell functions, the influence of ROS, DJ-1, Parkin, and neurotoxins were modeled. The behavior of the model was investigated using FBA. We showed that an increased DA concentration in the cell produces more ROS but does not induce cell death. An increased *α*SYN input flux induces ROS production, mitophagy, and finally apoptosis. Additionally, we observed that *α*SYN was permanently transported out of the cell. The cellular death induced by the neurotoxin MPTP was also investigated. The results confirmed that the cell model shows the same behavior as a normal nerve cell.

The developed SBML model is a core model of a dopaminergic cell, which contains the essential cellular processes. It can be easily extended with further experimental knowledge and assumptions. Thus, the model presented in this work can be used as a basis to conduct further *in silico* studies and to investigate PD in more detail.

## Methods

We developed six different experiments for the identification of steady model states and applied several FBAs with different target functions. Each experiment simulated different cell states and was performed using the simulation tool VANTED (version 2.01, [52]) and the add-on FBA-Sim Vis [53]. VANTED is an application for visualizing and investigating graphs and biological networks. Further, VANTED offers the possibility to investigate these networks statistically [52]. The VANTED add-on FBA-Sim Vis enables the performance of FBAs and the dynamic visualization of the resulting fluxes. The fluxes are calculated using the COBRA toolbox [54] and an adapted version of the Clp simplex solver [53].

### Flux balance analyses

We performed six different experiments with the application VANTED including the add-on FBA-Sim Vis: (i) investigation of the normal cell behavior, (ii) increase of O_2_, (iii) increase of ATP, (iv) influence of neurotoxins, (v) increase of *α*SYN in the cell, and (vi) increase of dopamine synthesis (see Table 1).

Bounds for each flux must be defined prior to performing an FBA. We took the flux values from Best *et al.* with a range of ± 10 % of the original values for the DA synthesis, metabolism, and transport [11] (see Additional files 3 and 4). Since the complete dopaminergic nerve cell model contains more reactions than the model by Best *et al.*, the lower bound values of the reaction OMSynthesis1, OMSynthesis2, HVASynthesis3, and HVASynthesis4 need to be set to 0 in order to enable the performance of a complete FBA.

If it is well-known from experiments and literature that some reactions appear rarely or proceed slowly, the upper bound can be restricted to a maximum of 30 *μ*M·h^−1^. For all other reactions, a flux range between 0 and 100 *μ*M·h^−1^ was defined.

Besides the flux bounds, it is necessary to define those reactions that should be minimized or maximized. These fluxes are also called target functions. In our experiments, we always minimized or maximized the apoptosis or degradation fluxes. The experiments and the corresponding fluxes are listed in Table 1.

## Abbreviations

DA: Dopamine; DAT: Dopamine transporter; FBA: Flux balance analysis; LB: Lewy body; PD: Parkinson’s diasease; MAO: Monoamine oxidase; MPTP: 1-methyl-4-phenyl-1,2,3,6-tetrahydropyridine; ROS: Reactive oxygen species; TH: Tyrosine hydroxylase; SBML: Systems Biology Markup Language; αSYN: *α*-synuclein; UPS: Ubiquitin-proteasome-system.

## Competing interests

Authors declare no competing personal or financial interests.

## Authors’ contributions

FB and AD conceived and designed the study. FB coordinated the study. SS and SH built the model, performed the flux balance analysis and the clustering. FB and CW annotated the model. SS, FB, SH and PJK planned the experiments. FB and PJK provided scientific background for model construction. AZ and PJK supervised the work. All authors read and approved the final manuscript.

## Supplementary Material

Additional file 1 **SBML model of the dopaminergic nerve cell.** The SBML file contains the annotated SBML model of the dopaminergic nerve cell. Click here for file

Additional file 2 **Reactions of the dopaminergic nerve cell model.** This table describes all reactions of the dopaminergic nerve cell model in detail with name and flux ranges. Click here for file

Additional file 3 **Entities of the dopaminergic nerve cell model.** This spreadsheet lists all model entities that are used in the dopaminergic nerve cell model. It also includes a description for each entity, as well as identifiers to commonly used databases. Click here for file

Additional file 4 **Comparison of the mathematical model by Best** ***et al.* to the dopamine sub-model of the dopaminergic nerve cell.** This file presents the comparison of the mathematical model by Best *et al.* to the sub-model of the dopaminergic nerve cell containing dopamine synthesis, metabolism, and transport. It lists in detail the adjusted flux ranges of the initial model and the result of the FBA of this sub-model. Click here for file

Additional file 5 **Detailed model of the dopaminergic nerve cell.** This file contains the visualization of the dopaminergic nerve cell model with its 139 reactions and 111 metabolites in detail. The metabolites are depicted as circles and the reactions as rectangles. Red circles visualize all reagents and products, whereas pink circles visualize units of currency. Reactions are colored blue. Reactions with the suffix ‘IN’ represent input reactions transporting metabolites in the cell (light green) and reactions with the suffix ‘OUT’ represent the corresponding output reactions (dark green). Click here for file
