# Supplementary material for: Parkinson’s disease: dopaminergic nerve cell model is consistent with experimental finding of increased extracellular transport of α-synuclein
Source: BMC Neurosci. 2013 Nov 6;14:136. doi: 10.1186/1471-2202-14-136 (PMC3871002; doi:10.1186/1471-2202-14-136)
Supplement: Additional file 2 — Reactions of the dopaminergic nerve cell model. This table describes all reactions of the dopaminergic nerve cell model in detail with name and flux ranges. [file 1471-2202-14-136-S2.pdf]

Parkinson's disease: dopaminergic nerve cell model is consistent with experimental finding of increased extracellular transport of α-synuclein

Finja Büchel*[1] , Sandra Saliger[1], Andreas Dräger[1,2], Stephanie Hoffmann[1], Clemens Wrzodek[1], Andreas Zell[1] and Philipp J. Kahle[3]

[1]Center for Bioinformatics Tuebingen (ZBIT), University of Tuebingen, 72076 Tübingen, Germany
[2]Bioengineering Department, University of California, San Diego, CA 92093-0412, USA
[3]Laboratory of Functional Neurogenetics, Department of Neurodegeneration, Hertie Institute for Clinical Brain Research and German Center for Neurodegenerative Diseases, University of Tuebingen, 72076 Tübingen, Germany

Email: Finja Büchel*- finja.buechel@uni-tuebingen.de;

*Corresponding author

Supplement 2 - Reactions of the dopaminergic nerve cell model

Reaction name	Flux value in [$\mu M \cdot h^{-1}$] Min.	Max.	Description
**Sub-model 1: Reactions of the dopamine synthesis:**			
eTYRIN	217	265	Input: extracellular tyrosine (precursor of the DA synthesis)
TYROUT	0	265	Storage pool of intracellular tyrosine
R_bh2_bh4_1	24.6	30	Side reaction of the conversion from tyrosine into L-DOPA
R_bh2_bh4_2	24.6	30	Side reaction of the conversion from tyrosine into L-DOPA
R_etyr_tyr_1	217	265	Transport reaction of tyrosine into the cytosol
R_etyr_tyr_2	217	265	Transport reaction of tyrosine into the cytosol
R_ldopa_cda_1	24.6	30	Conversion of L-DOPA to DA
R_ldopa_cda_2	24.6	30	Conversion of L-DOPA to DA
R_tyr_ldopa_1	24.6	30	Conversion of tyrosine into L-DOPA
R_tyr_ldopa_2	24.6	30	Conversion of tyrosine into L-DOPA
eda_TH_inhib	0	100	Inhibition of tyrosine through external DA
cda_TH_inhib	0	100	Inhibition of tyrosine through cytosolic DA
**Sub-model 2: Reactions of the dopamine transport:**			
eDAOUT	0.73	0.9	Output: external DA
R_cda_vda_1	72.9	89.1	Transport reaction of cytosolic DA into vesicles
R_cda_vda_2	72.9	89.1	Transport reaction of cytosolic DA into vesicles
R_da_transport	72.9	89.1	Transport reaction of DA (vesicle release)
R_eda_auto_1	0	100	Transport reaction of DA (automatical re-uptake)
R_eda_auto_2	0	100	Transport reaction of DA (automatical re-uptake)
R_eda_DAT_1	72.1	88.1	Transport reaction of DA (re-uptake using DAT)
R_eda_DAT_2	72.1	88.1	Transport reaction of DA (re-uptake using DAT)

Reaction name	Flux value in [$\mu M \cdot h^{-1}$] Min.	Max.	Description
**Sub-model 3: Reactions of the dopamine metabolism:**			
AcetaldehydIN	0	100	Input: acetaldehyde (metabolite of the salsolinol synthesis)
DOPACSynthesis1	0	100	Conversion of DOPAL into DOPAC
DOPACSynthesis2	0	100	Conversion of DOPAL into DOPAC
DOPALlikeROS	0	100	Effect of DOPAL as ROS
DOPALSynthesis1	1	10	Conversion of DA into DOPAL
DOPALSynthesis2	0	100	Conversion of DA into DOPAL
DopamineesyntheseROSin	1	15	ROS production through a side reaction with tyrosine
DopamineesyntheseROSout	0	100	ROS production through a side reaction with tyrosine
DOPETOUT	1	10	Output: DOPET (degradation product of the DA metabolism)
DOPETSynthesis1	0	100	Conversion of DOPAL into DOPET
DOPETSynthesis2	0	100	Conversion of DOPAL into DOPET
HVAOUT	1	15	Output: HVA (Degradation product of the DA metabolism)
HVAldehydSynthesis1	0	100	Conversion of 3-O-methyldopamine into HVAldehyde
HVAldehydSynthesis2	0	100	Conversion of 3-O-methyldopamine into HVAldehyde
HVASynthesis1	0	100	Conversion of HVAldehyde into HVA
HVASynthesis2	1	100	Conversion of HVAldehyde into HVA
HVASynthesis3	0	29.1	Conversion of DOPAC into HVA
HVASynthesis4	0	29.1	Conversion of DOPAC into HVA
OMSynthesis1	0	29.1	Conversion of DA into 3-O-methyldopamine
OMSynthesis2	0	29.1	Conversion of DA into 3-O-methyldopamine
SalsolinollikeROS	0	10	Effect of salsolinol as ROS
SalsolinolSynthesis	1	10	Conversion of DA into R-salsolinol
SNCADopamineAggregation	0	20	Reaction of DA and αSYN
THPOUT	1	10	Output: THP (degradation product of the DA metabolism)
THPSynthesis	0	100	Conversion of DA and DOPAL into THP
**Sub-model 4: MPTP reactions:**			
MitochondriaDamageMPP+	0	100	Damage of mitochondria with MPP+
MPTPIN	0	0	Input: MPTP (neurotoxin)
R_eMPP+_DAT_1	0	100	Transport reaction of MPP+ (uptake using DAT)
R_eMPP+_DAT_2	0	100	Transport reaction of MPP+ (uptake using DAT)
toxification1	0	100	Activation of MPTP to MPP+
toxification2	0	100	Activation of MPTP to MPP+
**Sub-model 5: Apoptosis reactions:**			
startApoptosis	0	100	Irreversible start of apoptosis
apoptosisLB	0	100	Apoptosis activated by LB
apoptosisMito	0	100	Apoptosis activated by mitochondria
cytC-transport	0	100	Side reaction of the apoptosis (cytochrome C release)
cytC-transport-inhibitionParkin1	0	10	Inhibition of the apoptosis (cytochrome C release)
cytC-transport-inhibitionParkin2	0	100	Inhibition of the apoptosis (cytochrome C release)
cytC-transport-inhibitionTRAP1-1	0	10	Inhibition of the apoptosis (cytochrome C release)
cytC-transport-inhibitionTRAP1-2	0	100	Inhibition of the apoptosis (cytochrome C release)
LBProduction	0	100	Formation of LB

Reaction name	Flux value in [$\mu M \cdot h^{-1}$] Min.	Max.	Description
initApoptosis	0	100	Origin of the apoptosis activated by mitochondria
stopApoptosis	0	100	Possibility of the cell to stop the apoptosis
**Sub-model 6: Degradation reactions:**			
degradationLysosom	0	30	Lysosomal degradation
degradationProteasome	0	30	Proteasomal degradation
degradedOUT	0	45	Overall reaction of the degradation
Mfn2IN	0	100	Input: Mfn2 (Metabolite of the mitophagy)
mitophagy	0	30	Degradation of defect mitochondria (mitophagy)
R1	0	100	Side reaction of the proteasomal degradation
R2	0	100	Side reaction of the proteasomal degradation
R4	0	100	Side reaction of the proteasomal degradation
R5	0	100	Side reaction of the proteasomal degradation
R6	0	100	Side reaction of the proteasomal degradation
RProteasomalDegradationStart	0	100	Side reaction of the proteasomal degradation
VDAC1IN	0	100	Input: VDAC (metabolite of the mitophagy)
**Sub-model 7: αSYN reactions:**			
isdamagedProtein	0	15	Effect as defect protein
MitochondriaDamageSNCA-Aggregates	0	15	Damage of the mitochondria by αSYN aggregates
SNCAIN	0	100	Input: αSYN
SNCAOUT	0	45	Output: αSYN
SNCAOverexpression	0	15	Formation of aggregates (increase αSYN level)
SNCAROSAggregation	0	30	Formation of aggregates (ROS)
**Sub-model 8: DJ-1 reactions:**			
DJ1IN	0	100	Input: DJ-1
fibrillationInhbitionDJ1-1	1	10	Inhibition of the formation of αSYN aggregates
fibrillationInhbitionDJ1-2	0	100	Inhibition of the formation of αSYN aggregates
ROSprotectionDJ1	0	10	Effect as antioxidant
**Sub-model 9: Reactions of the mitochondria:**			
MitochondriaDamageROS	0	50	Damage of the mitochondria by ROS
MitochondriaRepairp38	0	5	Repair/Inhibtion of defect mitochondria (p38)
MitochondriaDamageProteins	0	10	Damage of defect mitochondria by proteins
ROSprodMito	0	100	Mitochondria producing ROS
complexI	0	100	1st complex of the electron transport chain - Transfer of electrons from NADH to Ubiquinol
ROS_complexI	0	100	ROS producing equivalent of the 1st complex of the electron transport chain
complexII	0	100	2nd complex of the electron transport chain - Transfer of electrons from Succinate ($FADH_2$) to Ubiquinol
complexIII	0	100	3rd complex of the electron transport chain - Transfer of electrons from Ubiquinol to Ferrocytochrom C
ROS_complexIII	0	100	ROS producing equivalent of the 3rd complex of the electron transport chain
complexIV	0	100	4th complex of the electron transport chain - Transfer of electrons from Ferrocytochrom C to H_2O
ATPase	0	1000	Enzyme which converts the proton-gradient into ATP
protonPool	0	1000	Pool of protons needed for the proton-gradient

Reaction name	Flux value in $[\mu M \cdot h^{-1}]$ Min.	Max.	Description
energyCons	0	100	Substitutional reaction for ATP consuming processes in the cell
FumIN	0	100	Input: Fumarate
FumOUT	0	100	Output: Fumarate
SuccIN	0	100	Input: Succinate
SuccOUT	0	100	Output: Succinate
mtDNADamageROS	0	50	Damage of mitochondrial DNA by ROS
mtDNAIN	0	100	Input: mtDNA
newDefectMitochondria	0	100	Production of defect mitochondria
newViableMitochondria	0	100	Production of healthy mitochondria
p38IN	0	100	Input: p38
startBiogenesis	0	20	Start reaction of the production of new mitochondria
**Sub-model 10: Protein reactions:**			
HtrA2-PRepair1	0	5	Repair/Inhibtion of defect proteins (HtrA2)
HtrA2-PRepair2	0	100	Repair/Inhibtion of defect proteins (HtrA2)
HtrA2IN	0	100	Input: HtrA2
ParkinIN	50	100	Input: Parkin
ParkinOUT	0	45	Output: Parkin
PINK1IN	0	100	Input: PINK1
ProteinDamageROS	0	50	Damage of proteins by ROS
ProteinIN	50	100	Input: Protein
ProteinOUT	0	45	Output: Protein
proteinmisfolding-inhibition1	0	10	Repair/Inhibition of defect proteins (TRAP1)
proteinmisfolding-inhibition2	0	100	Repair/Inhibition of defect proteins (TRAP1)
R_Parkin_HtrA2_1	0	100	Activation of HtrA2 by PINK1
R_Parkin_HtrA2_2	0	100	Activation of HtrA2 by PINK1
R_Parkin_PINK1_1	0	100	Activation of Parkin by PINK1
R_Parkin_PINK1_2	0	100	Activation of Parkin by PINK1
R_Parkin_TRAP1_1	0	100	Activation of TRAP1 by PINK1
R_Parkin_TRAP1_2	0	100	Activation of TRAP1 by PINK1
TRAP1IN	0	100	Input: TRAP1
**Sub-model 11: Reactions of the units of currency:**			
ADPIN	0	1000	Input reaction: ADP+Pi
ADPOUT	0	100	Output reaction: ADP+Pi
ATPOUT	0	100	Output reaction: ATP
Fe2+IN	0	100	Input reaction: Fe^{2+}
Fe2+OUT	0	100	Output reaction: Fe^{2+}
Fe3+IN	0	100	Input reaction: Fe^{3+}
Fe3+OUT	0	100	Output reaction: Fe^{3+}
H2OIN	0	100	Input reaction: H_2O
H2OOUT	0	500	Output reaction: H_20
NAD+IN	0	100	Input reaction: NAD^+
NAD+OUT	0	100	Output reaction: NAD^+
NADHIN	0	100	Input reaction: NADH
NADHOUT	0	100	Output reaction: NADH
O2IN	0	100	Input reaction: O_2
O2OUT	0	100	Output reaction: O_2
