# Supplementary material for: Parkinson’s disease: dopaminergic nerve cell model is consistent with experimental finding of increased extracellular transport of α-synuclein
Source: BMC Neurosci. 2013 Nov 6;14:136. doi: 10.1186/1471-2202-14-136 (PMC3871002; doi:10.1186/1471-2202-14-136)
Supplement: Additional file 3 — Entities of the dopaminergic nerve cell model. This spreadsheet lists all model entities that are used in the dopaminergic nerve cell model. It also includes a description for each entity, as well as identifiers to commonly used databases. [file 1471-2202-14-136-S3.pdf]

Parkinson's disease: Investigation of a mathematical dopaminergic nerve cell model

Finja Büchel*[1] , Sandra Saliger[1], Andreas Dräger[1,2], Stephanie Hoffmann[1], Clemens Wrzodek[1], Andreas Zell[1] and Philipp J. Kahle[3]

[1]Center for Bioinformatics Tuebingen (ZBIT), University of Tuebingen, 72076 Tübingen, Germany
[2]Bioengineering Department, University of California, San Diego, CA 92093-0412, USA
[3]Laboratory of Functional Neurogenetics, Department of Neurodegeneration, Hertie Institute for Clinical Brain Research and German Center for Neurodegenerative Diseases, University of Tuebingen, 72076 Tübingen, Germany

Email: Finja Büchel*- finja.buechel@uni-tuebingen.de;

*Corresponding author

Supplement 3 - Entities of the dopaminergic nerve cell model

Metabolite name	Description	Identifier
3-O-Methyldopamine	4-(2-Aminoethyl)-2-Methoxyphenol	PubChem:1669
AADC	Aromatic L-amino acid decarboxylase	Entrez:1644
Acetaldehyde	Acetaldehyde	CHEBI:15343
ADP+Pi	Adenosine diphosphate and anorganic phosphate	CHEBI:16761
ALDH	Aldehyde dehydrogenase	EC:1.2.1.3
apoptoseFactors	factors of the apoptosis, such as cytochrome c or Smac/DIABLO	-
AR	Aldehyde reductase	EC:1.1.1.21
ATP	Adenosine triphosphate	CHEBI:15422
DRD2	D2 dopamine auto-receptor	Entrez:1813
bh2	Dihydrobiopterin	CHEBI:20680
bh4	Tetrahydrobiopterin	CHEBI:59560
BiogenesisMitochondria	Pseudo metabolite for the biogenesis of mitochondria	-
cda	Cytosolic dopamine	CHEBI:18243
cMPP+	1-methyl-4-phenylpyridinium-ion (cytosolic)	CHEBI:641
COMT	Catechol-O-methyltransferase	EC:2.1.1.6
cytC_Cyto	Cytochrome C (cytosolic)	Entrez:54205
cytC_IMS	Cytochrome C (inter membrane space)	Entrez:54205
damagedProtein	Damaged proteins	-
DAT	Dopamine transporter	Entrez:6531
degraded	Degradation products	-
DJ1	DJ-1	Entrez:11315
DOPAC	3,4-dihydroxyphenylacetic acid	CHEBI:41941
DOPAL	3,4-dihydroxyphenylacetaldehyde	CHEBI:27978
DOPET	3,4-dihydroxyphenylethanol	PubChem:82755
DRR	Dihydrobiopterin reductase	EC:1.5.1.34
DUB	Deubiquitinating Enzyme (UCH-L1)	Entrez:7345
E1	Ubiquitin-activating enzyme	Entrez:55236
E2	Ubiquitin-conjugating enzyme	Entrez:7319
E3Parkin	E3-Ligase (Parkin)	Entrez:5071

Metabolite name	Description	Identifier
eda	Extracellular dopamine	CHEBI:18243
eMPP+	1-methyl-4-phenylpyridinium-ion (extracellular)	CHEBI:641
etyr	Extracellular tyrosine	CHEBI:17895
Fe2+	Iron-2+ ion	CHEBI:29033
Fe3+	Iron-3+ ion	CHEBI:29034
Ferricyt_c	Ferricytochrome c	CHEBI:15991
Ferrocyt_c	Ferrocytochrome c	CHEBI:16928
Fumarate	Fumarate	CHEBI:18012
H2O	Water	CHEBI:33813
HtrA2	HtrA2	Entrez:27429
HtrA2-P	Phosphorylated HtrA2	Entrez:27429
HVA	Homovanillic acid	CHEBI:545959
HVAldehyde	Homovanillic aldehyde	CHEBI:545959
LBAggregates	Lewy bodies	-
ldopa	L-3,4-dihydroxyphenylalanine (L-DOPA)	CHEBI:15765
MAO	Monoamine oxidases	Entrez:4128
MAT	Monoamine transporters	-
Mfn2	Mitofusin-2	Entrez:9927
MPTP	1-methyl-4-phenyl-1,2,3,6-tetrahydropyridine	CHEBI:17963
mtDNA	Intact mitochondria DNA	-
mtDNADefect	Defect mitochondria DNA	-
NAD+	Nicotinamide adenine dinucleotide (oxidized)	CHEBI:13390
NADH	Nicotinamide adenine dinucleotide (reduced)	CHEBI:57945
O2	Oxygen	CHEBI:25806
p38	p38 mitogen-activated protein kinase	Entrez:1432
Parkin	Parkin	Entrez:5071
Parkin-P	Phosphorylated Parkin	Entrez:5071
PINK1	PTEN-induced putative kinase 1	Entrez:65018
PoolOfDefectMitochondria	Pool of defect mitochondria	-
PoolOfViableMitochondria	Pool of healthy mitochondria	-
Proteasome	26S-proteasome	-
Protein	Any protein	-
Protongradient	gradient established during the oxidative phosphorylation	-
R-salsolinol	R-salsolinol	PubChem:54456
ROS	Reactive Oxygen Species	CHEBI:18421
SNCA	alpha-synuclein	Entrez:6622
SNCAAggregate	alpha-synuclein aggregates	-
Substrate_Ubl_Ubl_Ubl	Polyubiquitinated substrate	-
Succinate	Succinate	CHEBI:15741
TH	Tyrosine hydroxylase	Entrez:7054
THP	Tetrahydropapaverolin	CHEBI:28770
TRAP1	TNF receptor-associated protein 1	Entrez:10131
TRAP1-P	Phosphorylated TRAP1	Entrez:10131
tyr	Tyrosine	CHEBI:17895
Ubl	Ubiquitin	Entrez:7314
Ubiquinol	Ubiquinol	CHEBI:17976
Ubiquinone	Ubiquinone	CHEBI:16389
vda	Vesicular dopamine	CHEBI:18243
VDAC1	Voltage-dependent anion-selective channel protein 1	Entrez:7416
vTyr	Vesicular tyrosine	CHEBI:17895
