# Supplementary material for: Parkinson’s disease: dopaminergic nerve cell model is consistent with experimental finding of increased extracellular transport of α-synuclein
Source: BMC Neurosci. 2013 Nov 6;14:136. doi: 10.1186/1471-2202-14-136 (PMC3871002; doi:10.1186/1471-2202-14-136)
Supplement: Additional file 4 — Comparison of the mathematical model by Best et al. to the dopamine sub-model of the dopaminergic nerve cell. This file presents the comparison of the mathematical model by Best et al. to the sub-model of the dopaminergic nerve cell containing dopamine synthesis, metabolism, and transport. It lists in detail the adjusted flux ranges of the initial model and the result of the FBA of this sub-model. [file 1471-2202-14-136-S4.pdf]

Parkinson's disease: Investigation of a mathematical dopaminergic nerve cell model

Finja Büchel*[1], Sandra Saliger[1], Andreas Dräger[1,2], Stephanie Hoffmann[1], Clemens Wrzodek[1], Andreas Zell[1] and Philipp J. Kahle[3]

[1]Center for Bioinformatics Tuebingen (ZBIT), University of Tuebingen, 72076 Tübingen, Germany
[2]Bioengineering Department, University of California, San Diego, CA 92093-0412, USA
[3]Laboratory of Functional Neurogenetics, Department of Neurodegeneration, Hertie Institute for Clinical Brain Research and German Center for Neurodegenerative Diseases, University of Tuebingen, 72076 Tübingen, Germany

Email: Finja Büchel*- finja.buechel@uni-tuebingen.de;

*Corresponding author

Supplement 4 - Comparison of the mathematical model from Best et al. to the sub-model of the dopaminergic nerve cell

The initial nerve cell model is built on the model information from Best *et al.* [1] containing the dopamine metabolism, synthesis and transport. Firstly, this model was transferred into the SBML format [2]. The fluxed of Best *et al.* were adjusted according to Table 4.1. with a range of ± 10 % of the original values to enable a flux balance analysis (FBA) solution in our basis model. We performed a FBA with the maximization of the external tyrosine (eTYRIN) reaction as target function. The resulting steady state fluxes are all at the lower bound of the ranges except for the tyrosine supplying reactions, which are maximized (see Table 4.1, *R_btyr_tyr_1* and *R_btyr_tyr_2*). Figure 4.1 shows the resulting flux map of the initial nerve cell model.

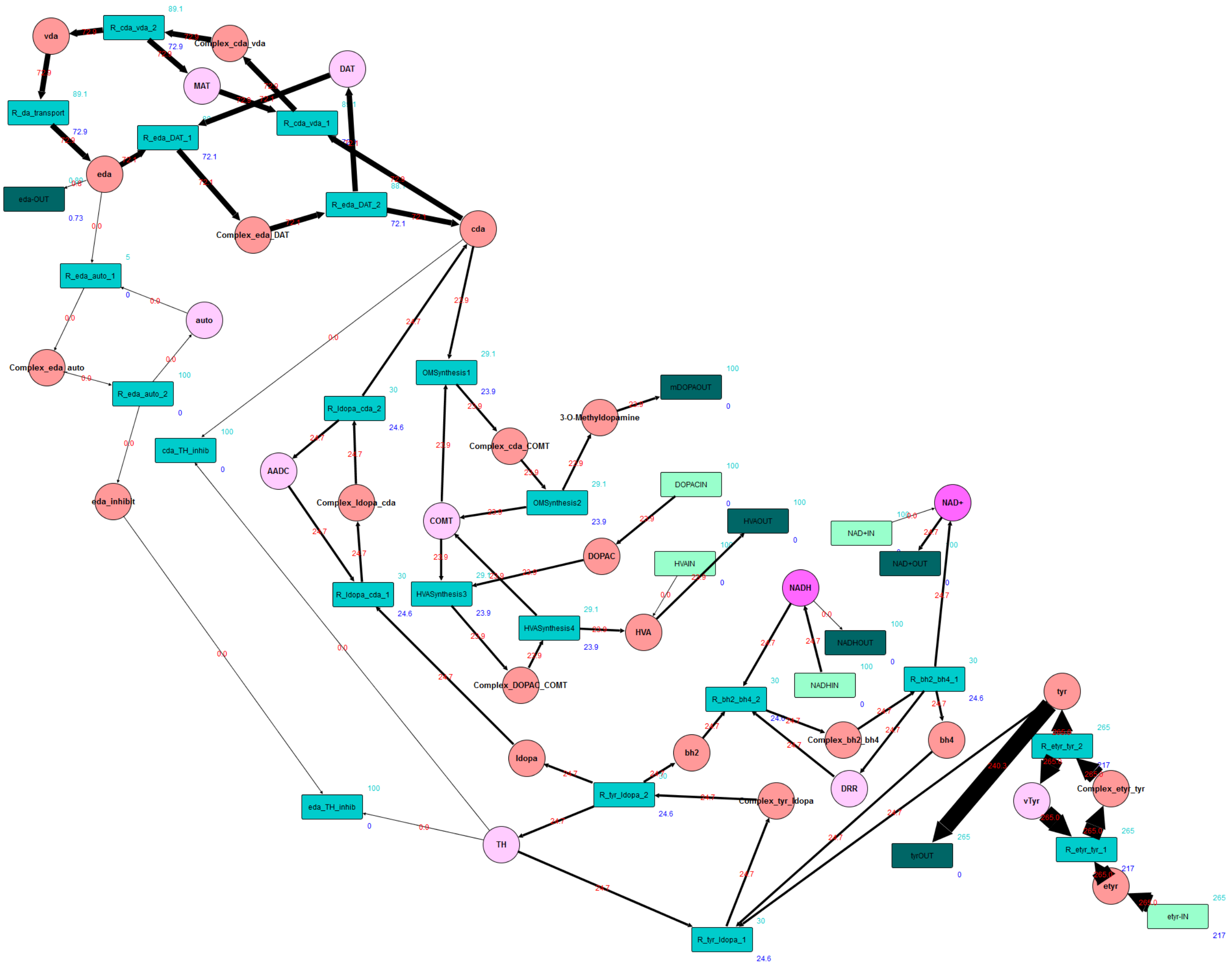

Figure 4.1: **Flux map of the mathematical model of Best *et al.*** Target function: maximization of the external tyrosine (eTYRIN). The majority of tyrosine is stored in the tyrosine pool (tyrOUT). The fluxes are adjusted with a range of ± 10% of the original model values.

Table 4.1 - Comparison of flux values

Reaction	Flux of Best *et al.* 2009 $[\mu M \cdot h^{-1}]$	Ranges $[\mu M \cdot h^{-1}]$	Flux of Büchel *et al.* 2013 $[\mu M \cdot h^{-1}]$	Deviation [%]
eTYRIN	241	217 - 265	265	110.0
eDAOUT	0.81	0.73 - 0.9	0.8	98.8
HVASynthesis3	26.5	23.9 - 29.1	23.9	90.2
HVASynthesis4	26.5	23.9 - 29.1	23.9	90.2
OMSynthesis1	26.5	23.9 - 29.1	23.9	90.2
OMSynthesis2	26.5	23.9 - 29.1	23.9	90.2
$R\_bh2\_bh4\_1$	27.3	24.6 - 30	24.7	90.5
$R\_bh2\_bh4\_2$	27.3	24.6 - 30	24.7	90.5
$R\_btyr\_tyr\_1$	241	217 - 265	265	110.0
$R\_btyr\_tyr\_2$	241	217 - 265	265	110.0
$R\_cda\_vda\_1$	81	72.9 - 89.1	72.9	90.0
$R\_cda\_vda\_2$	81	72.9 - 89.1	72.9	90.0
$R\_da\_transport$	81	72.9 - 89.1	72.9	90.0
$R\_eda\_DAT\_1$	80.1	72.1 - 88.1	72.1	90.0
$R\_eda\_DAT\_2$	80.1	72.1 - 88.1	72.1	90.0
$R\_ldopa\_cda\_1$	27.3	24.6 - 30	24.7	90.5
$R\_ldopa\_cda\_2$	27.3	24.6 - 30	24.7	90.5
$R\_tyr\_ldopa\_1$	27.3	24.6 - 30	24.7	90.5
$R\_tyr\_ldopa\_2$	27.3	24.6 - 30	24.7	90.5

References

1. Best JA, Nijhout HF, Reed MC: **Homeostatic mechanisms in dopamine synthesis and release: a mathematical model.** *Theor Biol Med Model* 2009, **6**:21, [http://dx.doi.org/10.1186/1742-4682-6-21].

2. Hucka M, Finney A, Sauro HM, Bolouri H, Doyle JC, Kitano H, Arkin AP, Bornstein BJ, Bray D, Cornish-Bowden A, Cuellar AA, Dronov S, Gilles ED, Ginkel M, Gor V, Goryanin II, Hedley WJ, Hodgman TC, Hofmeyr JH, Hunter PJ, Juty NS, Kasberger JL, Kremling A, Kummer U, Novère NL, Loew LM, Lucio D, Mendes P, Minch E, Mjolsness ED, Nakayama Y, Nelson MR, Nielsen PF, Sakurada T, Schaff JC, Shapiro BE, Shimizu TS, Spence HD, Stelling J, Takahashi K, Tomita M, Wagner J, Wang J, Forum SBML: **The systems biology markup language (SBML): a medium for representation and exchange of biochemical network models.** *Bioinformatics* 2003, **19**(4):524–531.
