# Supplementary figures and images for: Parkinson’s disease: dopaminergic nerve cell model is consistent with experimental finding of increased extracellular transport of α-synuclein
Source: BMC Neurosci. 2013 Nov 6;14:136. doi: 10.1186/1471-2202-14-136 (PMC3871002; doi:10.1186/1471-2202-14-136)

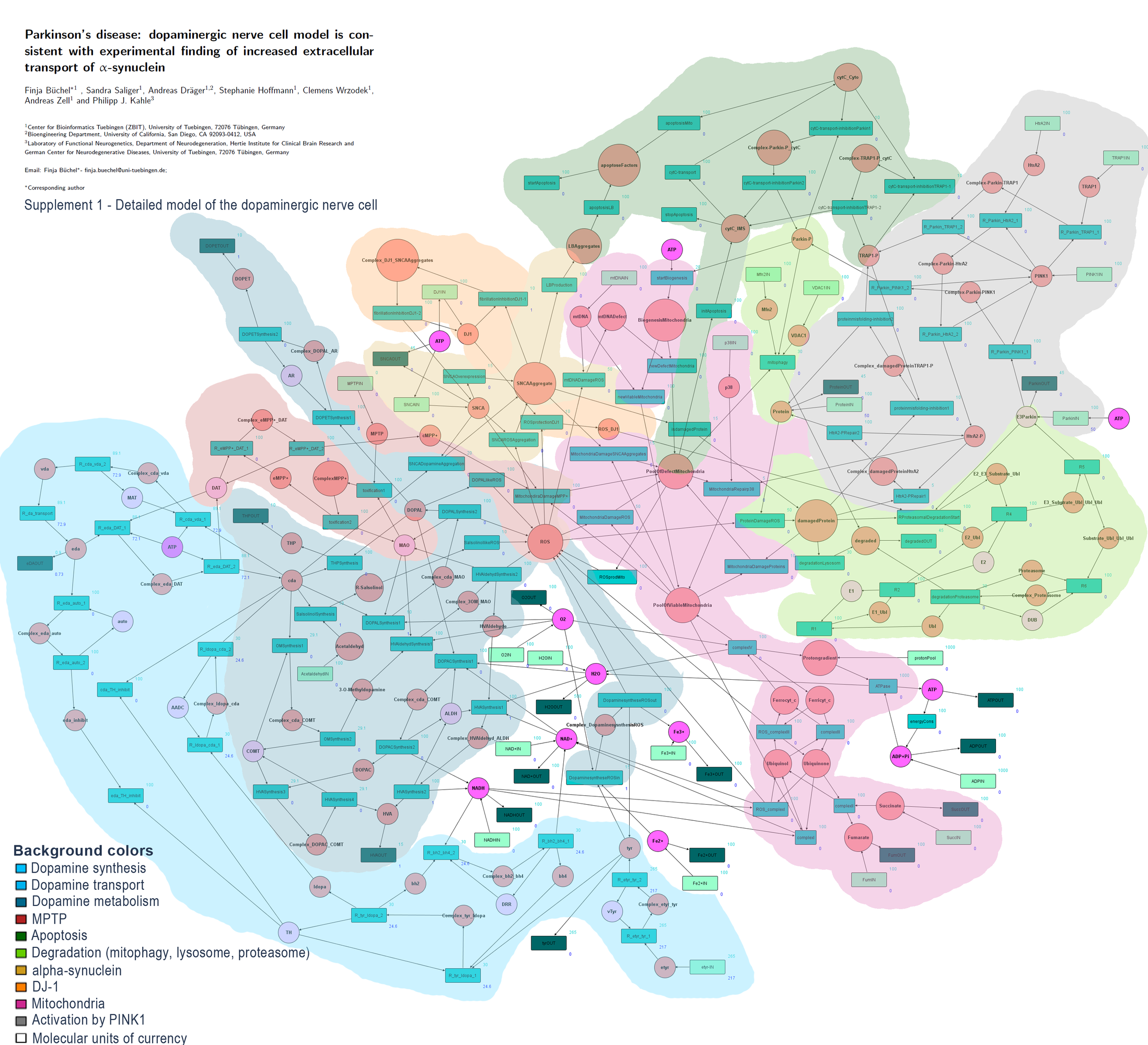

Supplement: Additional file 5 — Detailed model of the dopaminergic nerve cell. This file contains the visualization of the dopaminergic nerve cell model with its 139 reactions and 111 metabolites in detail. The metabolites are depicted as circles and the reactions as rectangles. Red circles visualize all reagents and products, whereas pink circles visualize units of currency. Reactions are colored blue. Reactions with the suffix ‘IN’ represent input reactions transporting metabolites in the cell (light green) and reactions with the suffix ‘OUT’ represent the corresponding output reactions (dark green). [file 1471-2202-14-136-S5.tiff]
